# Amino Acids Whose Intracellular Levels Change Most During Aging Alter Chronological Life Span of Fission Yeast

**DOI:** 10.1093/gerona/glaa246

**Published:** 2020-11-03

**Authors:** Charalampos Rallis, Michael Mülleder, Graeme Smith, Yan Zi Au, Markus Ralser, Jürg Bähler

**Affiliations:** 1 Institute of Healthy Ageing, Department of Genetics, Evolution & Environment, University College London, UK; 2 The Francis Crick Institute, London, UK; 3 Department of Biochemistry, Charité Universitaetsmedizin, Berlin, Germany

**Keywords:** aspartate, glutamine, metabolome, protein kinase A, *S pombe*

## Abstract

Amino acid deprivation or supplementation can affect cellular and organismal life span, but we know little about the role of concentration changes in free, intracellular amino acids during aging. Here, we determine free amino acid levels during chronological aging of nondividing fission yeast cells. We compare wild-type with long-lived mutant cells that lack the Pka1 protein of the protein kinase A signalling pathway. In wild-type cells, total amino acid levels decrease during aging, but much less so in *pka1* mutants. Two amino acids strongly change as a function of age: glutamine decreases, especially in wild-type cells, while aspartate increases, especially in *pka1* mutants. Supplementation of glutamine is sufficient to extend the chronological life span of wild-type but not of *pka1Δ* cells. Supplementation of aspartate, on the other hand, shortens the life span of *pka1Δ* but not of wild-type cells. Our results raise the possibility that certain amino acids are biomarkers of aging, and their concentrations during aging can promote or limit cellular life span.

Dietary restriction extends life span and decreases age-related pathologies (1,2). These benefits may reflect protein or amino acid restrictions rather than overall calorie intake (3,4). Restriction or supplementation of certain amino acids affects life span from yeast to mouse (5). In budding yeast, isoleucine, threonine, and valine extend the chronological life span (CLS, the time postmitotic cells remain viable in stationary phase) (6). In other yeast strains, serine, threonine, and valine decrease the CLS (7), while removal of asparagine extends the CLS (8). In mice, life span is extended by the branched chain amino acids (leucine, isoleucine, and valine) (9), while in flies, limitation of branched chain amino acids extends life span in a dietary-nitrogen-dependent manner (10). In mice, a tryptophan-restricted diet extends life span (11), while a methionine-restricted diet extends life span and delays age-related phenotypes (12). In flies, methionine restriction extends life span (13). Similarly, in budding yeast, methionine restriction prolongs the CLS while methionine addition shortens it (14). A comprehensive analysis of amino acid effects on life span has been undertaken in worms, where life span extends upon individual supplementation of 18 amino acids (15). These results indicate that amino acids can exert both pro- and antiaging effects. In budding yeast, intracellular amino acid content gradually decreases during chronological aging (16). What is missing, however, is a global quantitative analysis of intracellular amino acids in normal and long-lived cells during cellular aging.

In fission yeast (*Schizosaccharomyces pombe*), the protein kinase A (PKA) glucose-sensing pathway promotes chronological aging (17) but is not directly involved in amino acid sensing or transport (18). Here we perform quantitative amino acid profiling during aging in wild-type and long-lived *pka1* mutant cells of *S pombe*, which are deleted for the catalytic subunit of PKA. We show that intracellular amino acid pools progressively decrease with age. This effect is less pronounced in long-lived cells. Glutamine is depleted faster than the other amino acids, while aspartate increases during aging. Notably, supplementation of these 2 amino acids is sufficient to alter life span in wild-type or *pka1* mutant cells. Our results suggest both a correlative and causal relationship between longevity and intracellular amino acids.

## Method

### Strains and Media

Strain *972 h*^*−*^ was used as the reference wild-type. The *pka1* deletion mutant (*pka1::kanMX4 h*^*−*^) was generated with standard methods (19). Strains were cultured in EMM2 minimal medium for mass spectrometry sample acquisition, chronological aging, and amino acid supplementation experiments. Liquid cultures were grown at 32°C with shaking at 170 rpm.

### Quantitative Amino Acid Profiling

Amino acid analysis was performed using hydrophilic interaction chromatography-tandem mass spectrometry (HILIC-MS/MS) as described (20). Cell numbers were determined with a Beckman Z-series coulter counter to ensure equal cell amounts for extractions. The metabolites were extracted as described (20). Identification of 19 proteogenic amino acids was obtained by comparison of retention time and fragmentation patterns with commercial standards. Comparison against a standard curve produced from serial dilution of these standards allowed quantification of the free amino acids. The analysis was undertaken using an Agilent Infinity 1290 LC system with ACQUITY UPLC BEH amide columns (Waters Corporation, Manchester, United Kingdom) (pore diameter 130 Å, particle size 1.7 μm, internal diameter 2.1, column length 100 mm) coupled to an Agilent 6400 Series Triple Quadrupole LC/MS mass spectrometer operating in selected reaction monitoring mode. Cysteine was excluded from analysis due to its low stability. The co-eluting isomers threonine and homoserine were de-convolved using the homoserine-specific transition (m/z of 120->44).

### Amino Acid Supplementations

Aging cells were incubated in media containing amino acid supplements at Days 1 and 5, by removing the supernatant after centrifugation (1000 rpm, 3 minutes) followed by resuspension in EMM2, EMM2 with 20 mM glutamine, or EMM2 with 20 mM aspartate up to their original culture volume. Amino acids were not directly added to medium because of solubility issues. Supplementation happened specifically on the designated time points and not throughout the chronological aging.

### CLS Assays

Chronological life span assays were performed as described (21). Error bars represent standard deviations (of 9 measurements), calculated from 3 independent cell cultures, with each culture measured 3 times at each time point. Areas under the curve were measured for all experimental repeats with ImageJ (22). We compared areas under the curve for the portion of life-span curve after amino acids supplementation as before this time life-span curves were identical.

### Total Protein Measurements

We collected 10-mL liquid cultures during exponential growth (OD_600_ = 0.5–1.0), the start of stationary phase (Day 0), and after amino acid supplementation (Day 5). Cells were lysed using a Fastprep-24 (6.5 m/s, 60 seconds) with addition of 300-μL lysis buffer and 0.5-mm diameter glass beads. Protein concentrations were quantified by a Pierce BCA Protein Assay Kit following the manufacturer’s protocol, and absorbance values were obtained using the Magellan Data Analysis software. Proteins were separated in 4%–12% Bis-Tris gel (NuPAGE), and protein bands were detected using Ponceau S staining solution (Sigma–Aldrich, UK, P7170).

## Results

### Quantitative Amino Acid Analysis During Cellular Aging

**Figure 1.**
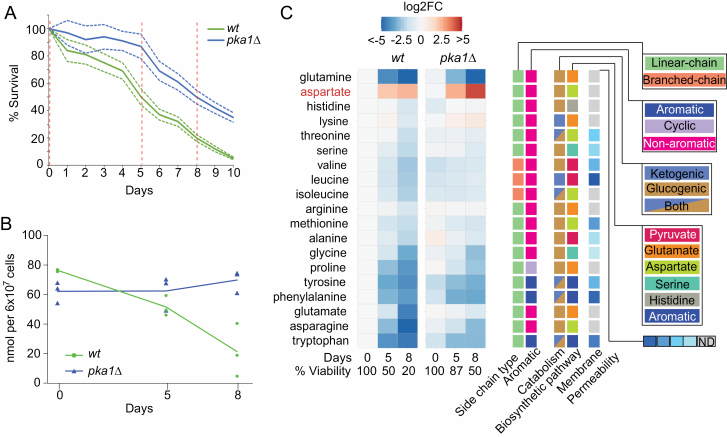
Quantitative measurements of free, intracellular amino acids in chronologically aging cells. (**A**) Chronological life span (CLS) assays for wild-type and *pka1Δ* cells, performed in triplicate with each biological replicate measured in 3 technical repeats. Average life spans are shown (solid lines) together with standard deviations (dotted lines). Hatched vertical lines indicate time points for amino acid profiling. (**B**) Total amino acid concentrations in wild-type and *pka1Δ* cells at different aging time points as indicated. (**C**) Heatmap showing quantitation of individual amino acids, along with known amino acid properties at right. The log2 fold-change values relative to 100% viability of wild-type cells (Day 0) were clustered using the R package pheatmap with standard settings. Averages of 3 time points with 3 independent biological repeats each are shown.

We used liquid chromatography-selective reaction monitoring (18) to quantify the free, intracellular pools for 19 of the 20 canonical proteogenic amino acids (cysteine was excluded as it is readily oxidized) during chronological aging of *S pombe* wild-type and long-lived *pka1Δ* deletion mutant cells. Cells were grown in minimal medium, because rich medium contains variable amounts of amino acids and cells accumulate intracellular amino acids when grown in rich medium (23). We collected cells from 3 independent biological experiments at 3 time points each: at Day 0, when cells enter stationary phase and both strains show 100% cell viability; at Day 5, when wild-type and *pka1Δ* strains show 50% and 87% viability, respectively; and at Day 8, when wild-type and *pka1Δ* strains show 20% and 50% viability, respectively (Figure 1A).

**Figure 2.**
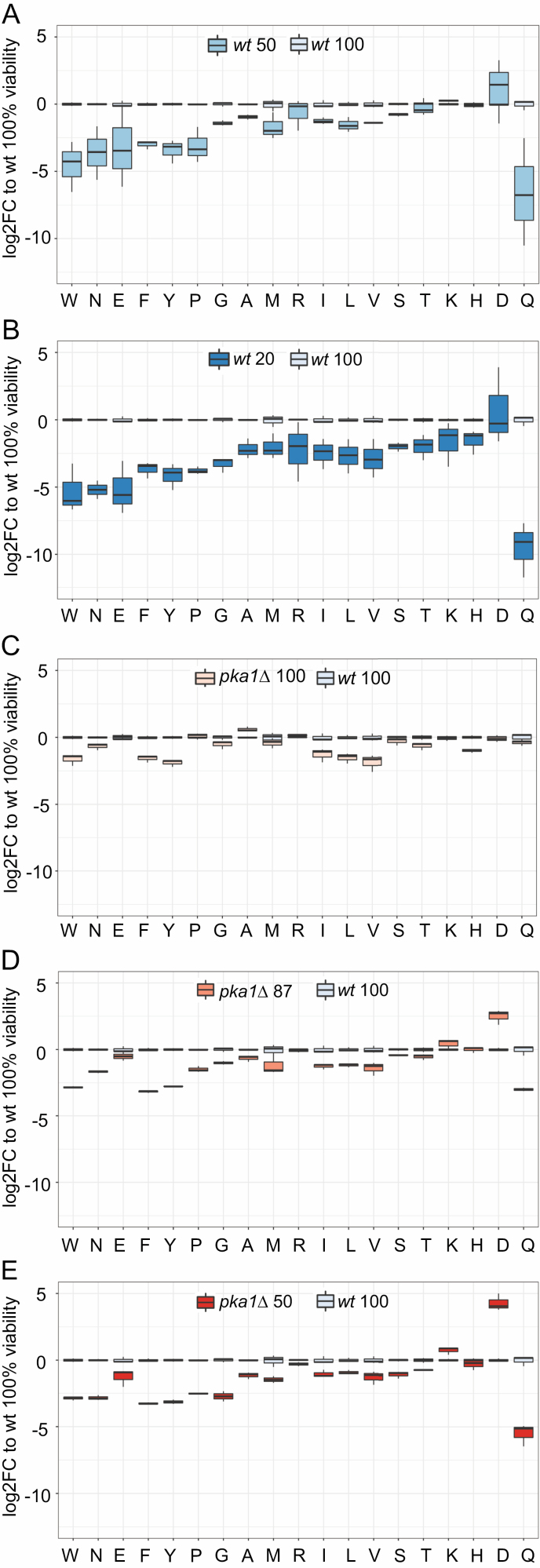
Changing amino acid concentrations during aging. Normalized concentration values of 19 amino acids in wild-type cells (panels A, B) and *pka1Δ* cells (panels C, D, E) during aging (top to bottom). For each condition, 3 independent samples were analyzed. Concentrations are presented relative to mean value for given amino acid in wild-type cells at 100% viability (wt 100). Boxplots were created with R boxplot and default settings. Numbers next to strain at top of each graph represent % cell viability.

We determined absolute concentrations of intracellular amino acids in nmol/6 × 10^7^ cells (Supplementary Table 1). The amino acid quantities, obtained from nondividing cells, differed from those previously reported for fission yeast which have been obtained from rapidly proliferating cells (20,24). At the onset of stationary phase, the total amino acid concentrations were lower in *pka1Δ* than in wild-type cells (Figure 1B). Accordingly, the individual amino acid concentrations were lower or similar in *pka1Δ* than in wild-type cells at that stage (Figures 1C and 2). The concentration differences were particularly pronounced for the branched chain amino acids and aromatic amino acids (phenylalanine, tryptophan, tyrosine) (Figures 1C and 2).

During chronological aging, the concentration of total free amino acids declined in wild-type but less so in *pka1Δ* cells, even rising slightly at the last time point (Figure 1B). This rise primarily reflected an increase in the most abundant amino acid, lysine, which compensated for the decline in other amino acids (Figure 2). In wild-type samples, the variation in free amino acids between experimental repeats noticeably increased during aging, in contrast to *pka1Δ* samples (Figures 1B and 2). This result raises the possibility that increased variation of amino acid concentrations, reflecting less tight metabolic regulation, is a feature of wild-type aging cultures.

Wild-type cells showed a general decrease in amino acids during aging, apart from aspartate (Figures 1C and 2). The branched chain amino acids were initially present at ~3-fold higher levels in wild-type cells before dropping to about half the level of *pka1Δ* cells (Figures 1C and 2). No correlations were evident between aging-dependent concentration changes of amino acids and their chemical/physical features or metabolic pathways (Figure 1C). This result suggests that changes in amino acid concentrations are not driven by limitation of precursor molecules. Likewise, there was no clustering based on glucogenic amino acids, which can be converted into glucose through gluconeogenesis (Figure 1C). This result suggests that any need for gluconeogenesis under glucose depletion does not greatly affect free amino acid composition. Reassuringly, there was also no clustering based on membrane permeability (Figure 1C), as this suggests that the results were not biased by amino acids leaking from nonviable cells during the aging time course.

Notably, the amino acid profiles of wild-type and *pka1Δ* cells were similar overall at corresponding aging time points, when both strains showed 50% viability (Figures 1B, C, and 2). This result suggests that similar metabolic changes occur in aging wild-type and mutant cells, but that these changes are delayed in the long-lived mutant cells. Some amino acids, however, showed distinct patterns in wild-type and *pka1Δ* cells, including lysine, glutamate, glutamine, and aspartate (Figures 1C and 2). Glutamine and aspartate showed particularly striking profiles during aging. Glutamine rapidly and strongly decreased during aging, more pronounced in wild-type cells (Figures 1C and 2). Aspartate, on the other hand, was the only amino acid that increased during aging in wild-type cells, and this increase was more pronounced in *pka1Δ* cells (Figures 1C and 2). These results pointed to glutamine and aspartate as markers for aging in *S pombe* and raised the possibility that these amino acids directly contribute to cellular life span.

### Glutamine Supplementation Extends Life Span of Wild-Type Cells

**Figure 3.**
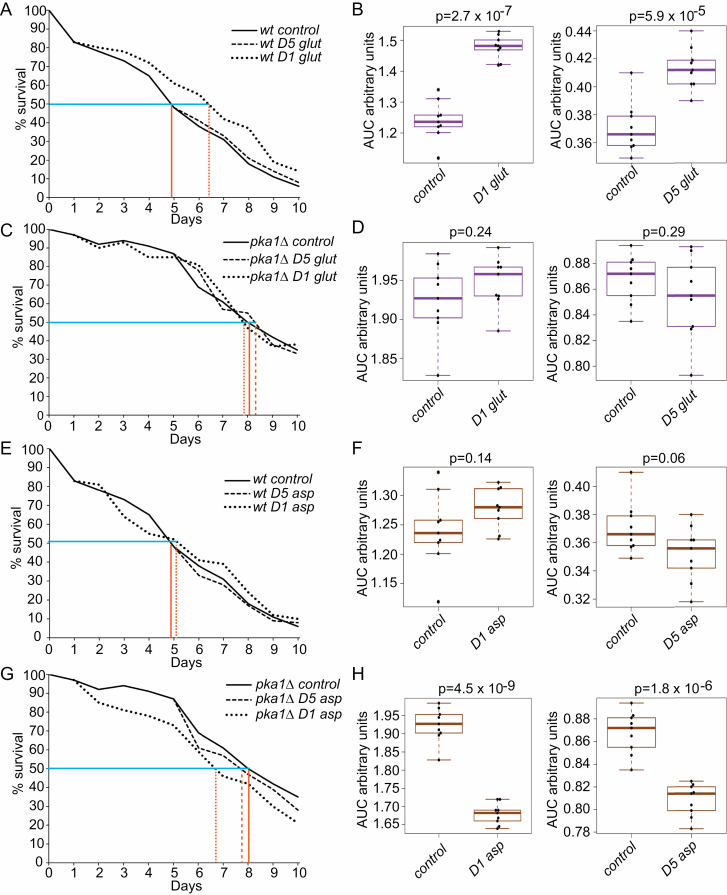
Effects of amino acid supplementation on chronological life span (CLS). (**A**) CLS assays (average of 3 biological repeats with 3 technical repeats each) for wild-type cells with and without glutamine supplementation at Days 1 and 5 as indicated. Median CLS in control cells (indicated with vertical lines) and treated cells (indicated with dotted vertical lines) are shown. (**B**) Areas under curve (AUCs) of CLS assays in A as indicated (left panel: AUCs from Day 1; right panel: AUCs from Day 5). The *p*-values (*t* test) indicate significance of difference in CLS triggered by glutamine supplementation. (**C**) CLS assays as in A for *pka1Δ* cells with and without glutamine supplementation at Days 1 and 5. (**D**) AUC of CLS assays in C, as described in B. (**E**) CLS assays as in A for wild-type cells with and without aspartate supplementation at Days 1 and 5. (**F**) AUC of CLS assays in E, as described in B. (**G**) CLS assays as in A for *pka1Δ* cells with and without aspartate supplementation at Days 1 and 5. (**H**) AUC of CLS assays in G, as described in B.

To examine the effect of glutamine on the CLS of wild-type and *pka1Δ* cells, we grew cultures to stationary phase in EMM2 minimal medium. During chronological aging, we replaced the medium with either EMM2 (control) on Day 1 or with EMM2 plus glutamine on either Day 1 (viability 84% ± 8% for wild-type and 97% ± 9% for *pka1Δ*) or Day 5 (viability 50% ± 6% for wild-type and 87% ± 8% for *pka1Δ*). These manipulations did not affect cell numbers or total protein levels of the aging cultures (Supplementary Figure 1). In wild-type cells, glutamine supplementation significantly extended the CLS, both when added on Day 1 or 5 (Figure 3A and B). Glutamine supplementation at Day 1 extended the medial CLS from 5 to 6.5 days. Although Day 5 was close to the median life span of wild-type cells, glutamine still extended the CLS even at this late stage (Figure 3A and B). These results indicate that glutamine is beneficial for viability of aging wild-type cells. In *pka1Δ* cells, on the other hand, glutamine supplementation at either Day 1 or 5 had no effect on life span, with the median CLS remaining ~8 days (Figure 3C and D). We conclude that glutamine addition during cellular aging promotes longevity in wild-type cells, but not in the long-lived *pka1Δ* cells which maintain relatively higher glutamine levels during aging (Figure 2).

### Aspartate Supplementation Shortens Life Span of *pka1Δ* Cells

To examine the effect of aspartate on the CLS of wild-type and *pka1Δ* cells, we performed the same experiment as with glutamine, but supplementing aspartate to chronologically aging cultures. Again, these manipulations did not affect cell numbers or total protein levels of the aging cultures (Supplementary Figure 1). In wild-type cells, aspartate supplementation at either Day 1 or 5 had no effect on the CLS (Figure 3E and F). In *pka1Δ* cells, on the other hand, aspartate led to a significant shorter CLS, both when applied on Day 1 or 5 (median CLS shortened from 8 to 6.7 or 7.7 days, respectively); however, the median CLS remained longer than that of wild-type cells (Figure 3G and H). We conclude that aspartate addition during cellular aging has no effect in wild-type cells but shortens the CLS in *pka1Δ* cells where aspartate naturally strongly increases during aging (Figure 2).

## Discussion

We report intracellular amino acid concentrations in *S pombe* as a function of both chronological aging and genetic background. Our results show an overall decrease in amino acids during chronological aging, especially in wild-type cells. Such a decrease has also been observed in budding yeast (16). We cannot exclude the possibility that some dead cells contributed to these amino acid measurements, although amino acids with high membrane permeability do not increase with age and thus do not preferentially leak from dead cells. The amount and composition of most amino acids reflect the “biological age” of wild-type and long-lived *pka1Δ* cells, being similar in cells of similar viability rather than similar chronological age. Hence, 5-day-old wild-type cells feature a similar amino acid signature as 8-day-old *pka1Δ* cells, corresponding to the times when both strains show 50% viability. Such amino acid signatures might therefore serve as aging biomarkers for *S pombe*.

Glutamine and aspartate show the most distinct profiles during aging, with a strong decrease of glutamine, especially in wild-type cells, and a strong increase of aspartate, especially in *pka1Δ* cells. The antagonistic changes in glutamine and aspartate are probably linked. During glucose deprivation, yeast cells turn to glutamine and glutamate for energy by making aspartate via glutaminolysis (25). Increased aspartate levels in aging *pka1Δ* cells could reflect that long-lived cells feature more efficient glutaminolysis, although alanine, another product of glutaminolysis, did not show the same trend. Induced glutaminolysis in long-lived cells could explain why aspartate did not increase life span in *pka1Δ* cells which naturally feature high aspartate levels.

Glutamine supplementation promotes longevity of wild-type but not of long-lived *pka1Δ* cells. Aspartate supplementation, on the other hand, shortens the life span of *pka1Δ* but not of wild-type cells. These amino acids also affect life span in worms (15): glutamine at high doses extends life span but at a lower dose shortens life span, while aspartate shortens life span. Glutamine and aspartate both affect mitochondrial functions which might mediate their life-span effects. Glutamine, derived from the Krebs cycle metabolite alpha-ketoglutarate, is one of the amino acids recently shown to become limited when blocking respiration in fermentatively growing *S pombe* cells (21). Amino acid supplementations at a later time point (Day 5) have weaker effects on longevity, likely reflecting the lower viability of the cell population at that time which diminishes any beneficial. It is actually surprising that the late supplementations, when cells are aging, still have some effect on life span.

Further experiments will provide mechanistic insights into the roles of glutamine and aspartate during aging. Our results suggest that decreased glutamine levels during aging not only correlate with aging in wild-type and *pka1Δ* cells but directly contribute to aging, which can be “cured” by glutamine addition in wild-type cells. These findings highlight the metabolic complexity of aging and its relationship with nutrient-sensing pathways like PKA. Interestingly, decreased glutamine levels are associated with aging also in budding yeast, rats, and humans (16,26), suggesting that conserved cellular processes are involved in this phenomenon.

## Supplementary Material

glaa246_suppl_Supplementary_Appendix
