# Supplementary material for: Amino Acids Whose Intracellular Levels Change Most During Aging Alter Chronological Life Span of Fission Yeast
Source: J Gerontol A Biol Sci Med Sci. 2020 Nov 3;76(2):205–10. doi: 10.1093/gerona/glaa246 (PMC7812441; doi:10.1093/gerona/glaa246)
Supplement: glaa246_suppl_Supplementary_Appendix [file glaa246_suppl_supplementary_appendix.pdf]

Appendix

eFigure 1: No effects of amino acids on aging cell numbers or protein levels.

eTable 1: Quantitation of 19 free amino acids (nmol/6 x 10exp7 cells) during chronological aging of wild-type and *pka1* mutant cells.

eFigure 1

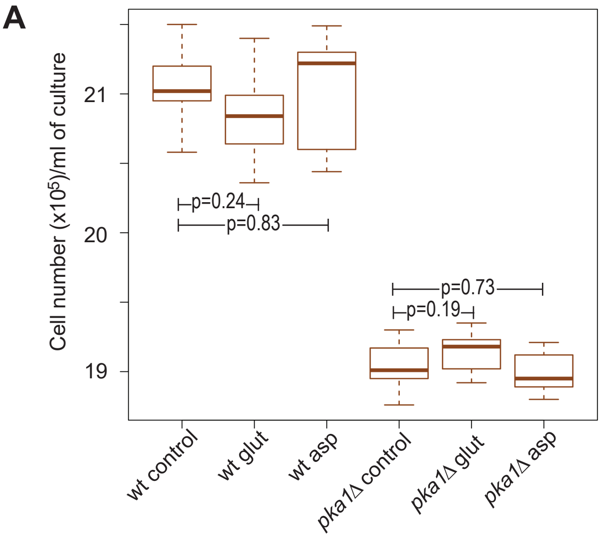

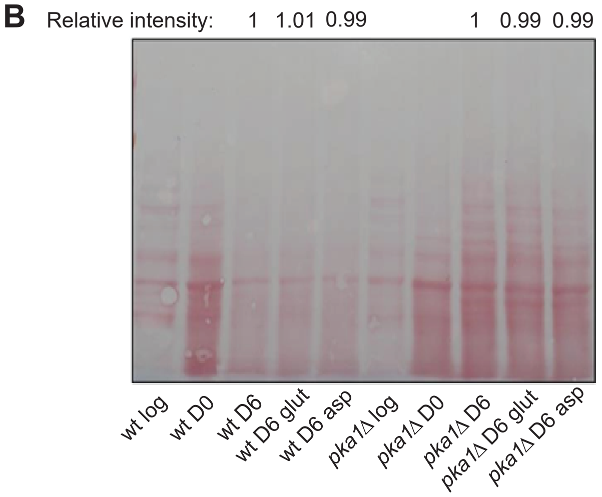

e Figure1. No effects of amino acids on ageing cell numbers or protein levels.
A. Cell numbers are unchanged within aged cultures following amino acid supplementation as indicated. Cell numbers were determined 24 hrs following change with media containing the supplemented amino acid.
B. Protein content is unchanged following amino acid supplementation as indicated.

eTable 1: Quantitation of 19 free amino acids (in nmol/6 x 10exp7 cells) during chronological aging of wild-type and *pka1* mutant cells

Sample	ala	asp	glu	phe	gly	his	ile	lys	leu	met	asn	pro	gln	arg	ser	thr	val	trp	tyr
**wt_100_A**	0.977	0.281	4.01	1.922	2.559	4.138	0.984	28.203	1.573	0.027	1.862	0.304	18.373	6.267	0.614	0.788	1.381	0.267	2.172
**wt_100_B**	0.994	0.289	3.956	1.958	2.537	4.619	1.003	27.09	1.637	0.018	2.07	0.307	18.779	6.078	0.664	0.866	1.499	0.291	2.342
**wt_100_C**	1.051	0.327	5.214	2.211	2.129	3.87	1.372	30.793	1.931	0.032	1.936	0.325	11.973	6.243	0.682	0.682	1.962	0.32	2.51
**wt_50_A**	0.585	2.875	4.251	0.294	1.039	3.927	0.564	32.32	0.881	0.017	0.623	0.096	2.833	7.224	0.398	0.452	0.603	0.041	0.355
**wt_50_B**	0.454	0.812	0.398	0.284	0.806	3.517	0.457	34.448	0.557	0.006	0.165	0.03	0.15	5.544	0.346	0.569	0.616	0.015	0.261
**wt_50_C**	0.526	0.11	0.062	0.198	0.881	3.969	0.403	35.722	0.414	0.004	0.04	0.016	0.011	1.585	0.393	1.058	0.621	0.003	0.11
**wt_20_A**	0.384	4.499	0.527	0.216	0.302	2.284	0.416	24.141	0.629	0.013	0.085	0.028	0.079	5.454	0.202	0.358	0.597	0.031	0.237
**wt_20_B**	0.203	0.248	0.091	0.188	0.315	1.876	0.221	13.038	0.276	0.005	0.033	0.022	0.03	1.594	0.128	0.218	0.208	0.004	0.154
**wt_20_C**	0.14	0.099	0.036	0.099	0.157	0.704	0.088	2.579	0.108	0.004	ND	0.019	0.005	0.257	0.171	0.098	0.083	0.003	0.063
**PKA1_100_A**	1.759	0.234	4.149	0.554	1.312	1.881	0.308	23.783	0.445	0.014	1.007	0.279	10.522	6.332	0.425	0.397	0.271	0.067	0.51
**PKA1_100_B**	1.376	0.331	5.04	0.752	1.883	2.277	0.595	29.602	0.736	0.025	1.417	0.378	13.836	7.233	0.599	0.556	0.627	0.117	0.692
**PKA1_100_C**	1.443	0.274	4.636	0.77	1.875	2.174	0.533	27.585	0.652	0.02	1.339	0.355	13.052	7.049	0.581	0.548	0.521	0.11	0.697
**PKA1_87_A**	0.687	2.227	3.085	0.237	1.106	4.547	0.506	46.875	0.792	0.009	0.655	0.106	2.034	5.373	0.485	0.567	0.684	0.041	0.352
**PKA1_87_B**	0.532	1.088	3.855	0.214	1.315	3.529	0.399	26.884	0.681	0.021	0.575	0.132	2.243	6.127	0.476	0.453	0.414	0.039	0.34
**PKA1_87_C**	0.728	1.968	2.464	0.233	1.196	4.554	0.504	44.549	0.802	0.008	0.628	0.098	1.884	5.885	0.504	0.603	0.802	0.043	0.342
**PKA1_50_A**	0.474	4.13	2.421	0.202	0.279	3.624	0.467	53.792	0.813	0.009	0.263	0.057	0.531	4.992	0.333	0.449	0.733	0.036	0.261
**PKA1_50_B**	0.37	9.541	1.084	0.208	0.473	2.517	0.487	38.026	1.011	0.011	0.245	0.052	0.186	5.488	0.251	0.468	0.444	0.042	0.239
**PKA1_50_C**	0.518	4.934	2.292	0.223	0.362	4.586	0.669	52.862	0.858	0.008	0.313	0.054	0.468	4.465	0.355	0.48	0.868	0.043	0.296
